# Reply to the Review of Dr. Eve’s Contribution on the History of Hip-joint Operations

**Published:** 1868-02

**Authors:** 


### Correspondence.

### Reply to the Review of Dr. Eve's Contribution on the History of Hip-joint Operations.

Mr. Editor:—A friend in Buffalo has kindly sent me your Journal for January, containing a notice of my contribution to the hip-joint operations performed during the late war in the Southern service. It is there stated I "rather exultingly compare" the result in the late Confederate army, with that published in Circular No. 6, from the Surgeon-General's Office of the United States; and moreover it is greatly regretted that one whose statements are so universally accepted, should have permitted himself to arrive at such premature conclusions, for that while his pamphlet was in construction, Circular No. 7, July, 1867, was issued by this same officer of government, which it is alleged contradicts my figures.

I respectfully solicit space in your Journal for the following remarks and corrections in reference to the insinuation above made touching my statements. I should not have troubled you, and would have permitted the spirit of criticism, emanating as it does from the old sectional feeling, which I had hoped was buried forever, especially in medicine, to pass unnoticed, but for the attack upon the veracity of my conclusions.

1st.—Let the readers of the *Buffalo Medical & Surgical Journal* know, a fact carefully concealed from them, that my investigations on the subject of the hip-joint operations were made at the special request, and for the use of the Surgeon-General of the United States Army; and that in this very Circular, No. 7, referred to, my contributions are acknowledged again and again, and my *co-operation* declared the most *cordial* and intelligent. Moreover, my article was accepted by the American Medical Association at its meeting last May in Cincinnati, and on its very cover bears its *imprimatur*.

2d.—It is not *true* that I stated Dr. Grant's case to be a successful primary amputation. Opposite to it in my statistics are the word, "result *doubtful.*" Drs. East's, Fauntleroy's, Compton's and Gilmore's, (not Gilman's as published in your Journal,) make *four*, without adding Dr. Grant's case.

3d.—I thought when Dr. Gilmore wrote that to sustain his patient he had even to send a daily messenger to Richmond, the capital of the then government, for necessary supplies, it was surely good evidence of the destitution in the South at that time. It will take stronger arguments than the reviewer urges to convince us that our poor wounded soldiers received all the *sanitary comforts* and *nutritious regimen* which they *required*, or that any class of them thus fared. We of the South still, and always will, believe that even those in health had a pretty hard struggle for life. The war certainly cost us something.

4th.—To make out an apparent contradiction in my report as to the success of the Federal service compared to the Southern, the reviewer *has actually taken one of my own cases*, Dr. Fauntleroy's, to make his seven recoveries. *Four*, too, of the seven, were *re*-amputations, the success in which no surgeon will admit now as a fair or true ratio in amputation at the hip-joint. Deduct these cases, and which every statistician knows is right and proper, and from the critic's own admission, he has only *three* successful in *thirty-four* coxo-femoral disarticulation; but putting it aside, the figures will read 3 in 20, with one doubtful on the Southern side, while on the Northern there were only 3 in 34. How, too, was I to know that in Circular No. 7, the three *re*-amputations would be added when this was published months after my report was issued from the press? I used one case of *re*-amputation in four successful, the reviewer four of this class in seven of his recoveries, one of them being mine. To this I object.

5th.—I stated that in carrying out the request of the Surgeon-General of the United States Army, the searcher after truth had been led "*unwittingly*" to a most favorable result on the side least expected, when we consider the destitute and isolated condition of the South during the war. This is the whole of my exultation over the comparison of the hip-joint operation in the two services. Unwittingly does not mean to *exult*.

I believe courtesy requires that when even a doubt is cast on an author's statements, the editor of the publication should at least notify him of it, that he may have space in the same paper for defense. May I request that you will be pleased hereafter to observe this good rule? Should you decline to publish this, do return it.

Respectfully, PAUL F. EVE.

Nashville, Tenn., Feb. 13, 1868.

---

RACINE, Wisconsin, Feb. 14th, 1868.

Editor Buffalo Medical and Surgical Journal:

*Dear Doctor:*—In the report of the proceedings of the Buffalo Medical Association for January, Prof. White gives a case in which he removed a child's diaper-pin from the rectum of a woman. Strange pin-cushion that! Dr. Boardman, a case where a nail, two and a half inches long, had been swallowed by a ruptured child, and which produced no unpleasant symptoms, but passed in due time. Also a case reported to him, of an adult, who disposed of a $20 gold piece in the same way. With Dr. Chapman we should of course expect that to pass, "if it was good." Doctor Strong, (no mis nomer, either,) calls up his case, reported many years ago, of a child two and a half years old, swallowing (with the Doctor's assistance,) an old-fashioned copper cent, "an inch and two lines in diameter, and two lines in thickness." This, too, "did get out, and after three or four weeks, passed per rectum."

Now, Mr. Editor, I can tell a larger story than either of these, or all of them tied up in a bunch, and just as true. In 1860, when practicing in Warsaw, Wyoming county, N. Y., I was sent for in great haste, to visit a child only two years of age, a boy, who had actually *swallowed his father and mother*, and if possible, stranger still, *both went down at the same time.* You can hardly imagine the consternation and horror written upon the countenances of all in the house, who had become acquainted with this sudden disappearance of both parents of the child. That they had been swallowed there was no doubt, for at least a dozen responsible persons testified to it at once. My countrymen!! wasn't I in a dilemma? How in the name of the father of medicine and surgery, was I to get them out? I had a little hope, at first, that the child might explode, and, thus at the expense of its own life, save father and mother; but no, it wouldn't go off. I debated in my own mind a few minutes, what course to pursue; but knowing both parents *as they went into the stomach* to be pretty *hard* customers, I had little fear of their digesting, for I felt quite sure they could stand confinement (not solitary) as long as Jonah did, so I began to breathe easy, and take time for a philosophical solution of the situation.

Now then for an explanation, which doubtless has already been anticipated by the reader. The patient had been left in charge of a nurse girl, who had given it, as a plaything, a double locket, containing a likeness of both its father and mother. The child, almost as soon as it came in possession of it, conveyed it to its mouth, and the girl becoming frightened in her attempt to get it out, actually thrust it down the throat into the œsophagus, where it remained nearly three hours, but it had passed completely into the stomach a few moments before I arrived, as I was distant about three miles from the patient, when sent for. The exact size of the locket, I cannot now state, but it was certainly as great in diameter as the old-fashioned cent, and of course much thicker. It remained eight days in the intestinal canal. Not an unpleasant symptom occurred during its stay. On the eighth day the parents became very anxious, (why should they not?) and from the operation of a very large dose of castor oil, which I had ordered, the locket passed.

I think Dr. Strong will cheerfully pass over the "laurels" to me, and call my case the most wonderful. For a father and mother to be swallowed by their own child, a little boy, of only two years of age, and to travel his entire length, through sphincter and all, in eight days, and to be safely deposited in a *pot-de-chambre*, is rather remarkable, even for this fast age, and a little beyond Dr. Strong's "copper."

I did not inquire of the "parents" after their second advent into this world, whether they took "notes by the way" or not, but if Dr. S. is not yet willing to pass over the laurels, I will do so, for they still live in glorious old New York.

JOHN G. MEACHEM.

